# Arthroscopic double-row rotator cuff repair: a comprehensive review of the literature

**DOI:** 10.1051/sicotj/2018048

**Published:** 2018-12-14

**Authors:** Nuri Aydin, Bedri Karaismailoglu, Mert Gurcan, Mahmut Kursat Ozsahin

**Affiliations:** Istanbul University-Cerrahpasa, Cerrahpasa Medical Faculty Istanbul Turkey

**Keywords:** Rotator cuff tear, Arthroscopic repair, Review, Surgical technique, Clinical outcome.

## Abstract

Rotator cuff repairs seek to achieve adequate tendon fixation and to secure the fixation during the process of biological healing. Currently, arthroscopic rotator cuff repair has become the gold standard. One of the earliest defined techniques is single-row repair but the inadequacy of single-row repair to precisely restore the anatomical footprint as well as the significant rates of retear especially in large tears have led surgeons to seek other techniques. Double-row repair techniques, which have been developed in response to these concerns, have various modifications like the number and placement of anchors and suture configurations.

When the literature is reviewed, it is possible to say that double-row repairs demonstrate superior biomechanical properties. In regard to retear rates, both double row and transosseous equivalent (TOE) techniques have also yielded more favorable outcomes compared to single-row repair. But the clinical results are conflicting and more studies have to be conducted. However, it is more probable that superior structural integrity will yield better structural and functional results in the long run. TOE repair technique is regarded as promising in terms of better biomechanics and healing since it provides better footprint contact. Knotless TOE structures are believed to reduce impingement on the medial side of tendons and thus aid in tendon nutrition; however, there are not enough studies about its effectiveness.

It is important to optimize the costs without endangering the treatment of the patients. We believe that the arthroscopic TOE repair technique will yield superior results in regard to both repair integrity and functionality, especially with tears larger than 3 cm. Although defining the pattern of the tear is one of the most important guiding steps when selecting the repair technique, the surgeon should not forget to evaluate every patient individually for tendon healing capacity and functional expectations.

## Introduction

The surgical treatment of rotator cuff tears, which can hinder the daily activities and the quality of life significantly and cause significant pain, has been practiced for years and improvements to surgical techniques are constantly achieved. Rotator cuff repairs seek to achieve adequate tendon fixation and to secure the fixation during the process of biological healing. Although various techniques like open surgery and mini open surgery have been frequently used in the past, currently, the use of arthroscopic rotator cuff repair has become the gold standard owing to the development of arthroscopic techniques. One of the earliest defined arthroscopic techniques is single-row repair but the inadequacy of single-row repair to precisely restore the anatomical footprint as well as the significant rates of retear especially in large and massive tears have led surgeons to seek other techniques [1,2].

Due to the belief that the restoration of rotator cuff anatomic footprint can lead to superior healing rates and functional outcomes, Fealy et al. [3] defined double-row anchor repair with mini open incision for the first time in 2002. The development of arthroscopic double-row rotator cuff repair, which was defined by Lo and Burkhart [4] in 2003, has opened a new era in the treatment of rotator cuff tears. The authors have proposed that with this technique, a wider restoration of the anatomic footprint could be achieved, the repair strength would be higher, there would be less stress on the anchors and knots and better healing rates could be achieved. However, since it is a more complicated and expensive surgery, questions regarding the cost effectiveness of double-row repairs were raised after some studies found similar functional outcomes of single-row and double-row techniques. In 2006, Park et al. [5] have defined the arthroscopic Transosseous Equivalent (TOE) technique, also known as the suture-bridge technique, based on the transosseous technique, which had been used in open surgery as gold standard. With this technique, the authors aimed to achieve a better healing process by providing better tendon bone contact and less trauma to the torn tendon.

Numerous surgical techniques and their modifications exist for the treatment of rotator cuff tears. Although the repair techniques differ with regard to anchor numbers, locations and suture configurations, all double-row repairs are technically more challenging, require additional anchors and are thus more expensive as well as more time-consuming procedures. That's why a surgeon should be well informed about the latest literature to apply the most appropriate and cost-effective technique in terms of better healing and functional outcomes. In this literature review, we aimed to present the proven or possible advantages and disadvantages of arthroscopic double-row rotator cuff repair and its main modifications in terms of biomechanical properties, healing capacity, rerupture rates and functional outcomes.

### Surgical technique

**Figure 1:**
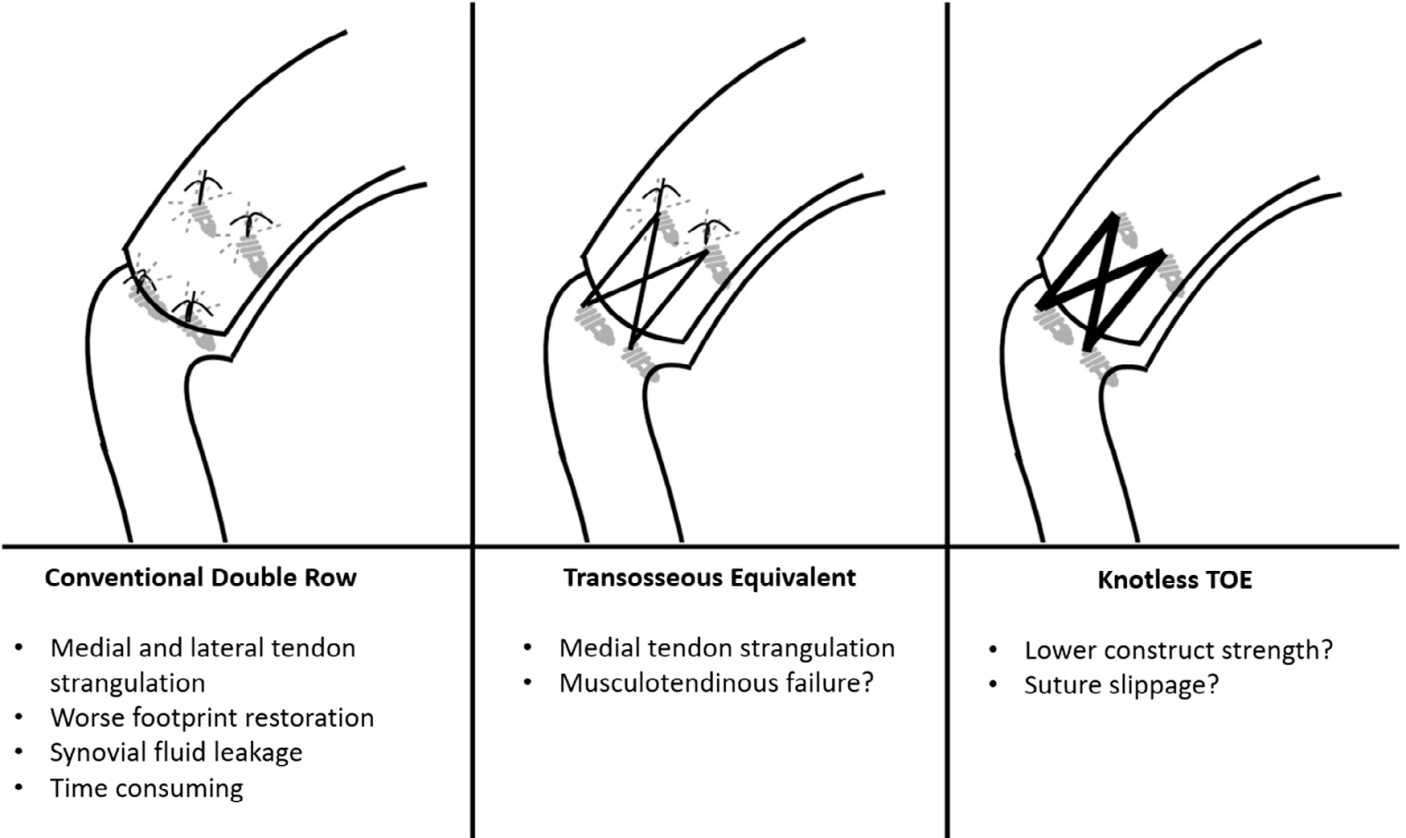
Main double-row repair constructs and their possible disadvantages.

Arthroscopic double-row rotator cuff repair is a structure that utilizes two rows of anchors, one being medial and the other being lateral, in order to provide better anatomical footprint restoration (Figure 1). In order for the repair structure to not lead to excessive tension, the surgeon should first free and mobilize the torn tendon and ensure that the tendon can reach the lateral side of the tuberculum majus. Double-row repair is more suitable for tears that can be reduced to the lateral of tuberculum majus without significant tension. In the conventional method, the medial row suture anchors are placed adjacent to articular margin first. Medial row sutures are passed through at least 10–12 mm medial to the lateral edge of the torn rotator cuff tendon in a horizontal mattress fashion. Subsequently, the lateral row suture anchors are placed along the lateral side of tuberculum majus. The sutures at the lateral row anchors are passed through the lateral side of the tendon by simple suture configuration and tied. After that, the medial row sutures are tied with proper tension. The preference about the number of the anchors can vary according to the size of the tear, but it should be ensured that there remain adequate blocks of bone between the anchors in order to prevent the risk of anchor overcrowding and anchor failure. While this technique allows for a wider contact area and a higher repair strength, it has disadvantages such as longer intraoperative time and the increase in cost stemming from the increase in anchor numbers. In addition, it is technically challenging and is associated with a steeper learning curve. Its other disadvantages include the overcrowding of anchors at the repair site, the inability of point fixations to provide sufficient compression at the rotator cuff footprint and the consequent inability to fully prevent synovial fluid leakage at the repair site [6,7].

Despite the superior biomechanical features and wider footprint restoration of the double-row repair, due to its failure to provide the desired degree of improvements regarding reruptures and clinical outcomes, it has been deemed cost-ineffective and other techniques have been sought out. The suture-bridge technique developed by Park et al. [5], otherwise known as the transosseous equivalent (TOE) technique since it was developed based on the transosseous technique used in open repair, has been regarded as promising in terms of biomechanical features (Figure 1). In contrast to conventional double-row repair, in the TOE technique, after the medial row has been placed and tied, the suture feet taken from the medial row are crossed over and passed from the interference screws which are placed 1 cm lateral to the footprint, compressing the tissue to the anatomical footprint. By this way, the tendon tissue isn't penetrated at the lateral row, tissue strangulation by the knots is decreased and tendon vascularity is probably better preserved [8]. With this technique, instead of the two-row point fixation of conventional double-row repair, the rotator cuff tissue is compressed to the anatomical footprint widely by the help of bridging sutures. The concern about anchor overcrowding is solved by sliding the second fixation row to the lateral of the tuberosity. In addition, there are fewer steps in the process of passing the sutures and there is less suture material placed between the tendon and the bone. As a conclusion, the operation involves fewer steps and the intraoperative time is reduced. This method is thought to aid in the rotator cuff healing process. In some studies, the musculotendinous junction has been pointed out as the primary failure point for double-row repair and TOE repairs [9,10]. The revision of failures seen at this point are difficult and for prevention, the medial row should not be over stretched and the medial row sutures should be placed lateral to the musculotendinous junction.

High rate of medial insufficiency in the conventional TOE repair, because of the excessive load and tendon strangulation at the medial knots, led surgeons to seek out preventive measures to improve the medial row integrity. In order to alleviate this concern, completely knotless TOE technique modifications have been defined [11] (Figure 1). In this technique, the sutures with wider surfaces are loaded to the medial row anchors and passed through the medial of the tendon without tying any knots, crossed over and fixed to the lateral row knotless anchors. This technique, which is technically simpler and carried out faster because of the lack of a need for knots, has been developed in order to eliminate the problem of tendon strangulation at the medial row knots and to reduce the increased cost related to prolonged intraoperative time. It is also aimed to reduce the increased suture-tendon surface pressure which could disrupt the healing potential.

### Biomechanics

Biomechanical tests and experimental models hold an important place in the first analysis of whether the developed rotator cuff repair techniques provide an advantage over previous techniques. Various cadaveric and animal models are frequently employed for this purpose. Although we cannot foresee the healing potential of the repair techniques in these experiments, questions such as how much fixation and failure strength the technique provides especially at the first moment of the repair, to what degree does the technique provide footprint restoration and whether it allows gap formation, can be demonstrated on a shoulder model and the biomechanical advantages and disadvantages that the techniques have in regard to each other can be shown. In conclusion, a technique must be biomechanically adequate in order to provide a good healing environment and provide adequate fixation until healing is accomplished. Many biomechanical studies have been conducted and are being conducted for this purpose.

The required biomechanical features that should be provided in a rotator cuff repair, as defined by Gerber et al. [12], are a high initial fixation strength, minimal gap formation and the continuation of mechanical stability until durable tendon-bone repair is completed. One of the most important areas where double-row repair is superior to single-row repair with a high level of evidence is biomechanics. Brady et al. [2] have shown that single-row repair doesn't cover 52.7% of the anatomical rotator cuff footprint and have proposed that this situation can lead to substantial morbidity. On the other hand, Meier and Meier [13] employed 3D mapping and demonstrated that double-row repair has the capability to restore the footprint as high as 100% and that this ratio was significantly higher than single-row or transosseous fixations. In the same study, they have shown that double-row repair reduces tendon–bone interface mobility more than single-row or transosseous repairs and that this could lead to a better healing environment. Milano et al. [14] have investigated the effect of tension on the reconstruction area on porcine cadavers and concluded that double-row repair is sturdier than single-row repair.

In contrast to these studies, some biomechanical studies have failed to denote a difference between double-row and single-row repairs. Mahar et al. [15] have found no demonstrable difference in terms of strength on bovine cadaveric shoulder model when they compared double and single-row repair techniques. Nelson et al. [16] have similarly found no difference between double- and single-row repair techniques under cyclic load.

Although there are some data suggesting that there may not be a difference in biomechanical features between these two techniques, most studies support that double-row repair has a stronger structure in rotator cuff repair [14,17,18]. Due to better restoration of the footprint, higher initial and failure strength, increased footprint contact pressure and lesser gap formation risk, it has been thought that double-row repair can lead to a better healing environment at the tendon–bone interface and enable more aggressive postoperative rehabilitation.

Upon the development and popularization of the TOE technique, biomechanical studies on this technique have also been more frequently done. Some fresh frozen cadaver studies have demonstrated that the TOE technique improves the contact features at the rotator cuff footprint, creates higher pressure at the tendon–bone interface and increases failure strength in comparison to conventional double-row repair [19,20]. In a study where Behrens et al. [21] have compared single-row repair and the TOE technique, they found no differences in terms of gap formation but found that the TOE technique has higher fixation strength. Nassos et al. [22] have shown on cadaveric models that the TOE technique prevents synovial fluid leakage at the rotator cuff footprint better than conventional double-row repair and proposed that this situation could provide a better healing environment.

Some researchers have evaluated the contribution of the medial row knots to biomechanical properties in double-row repair and suggested that the medial row knots reduces the failure load by preventing gap formation and absorbing the energy, and therefore preserve the rotator cuff footprint [23,24]. In a cadaveric study, Busfield et al. [23] have detected less gap formation and higher failure strengths in the group with medial knots. Pauly et al. [24] have shown that medial mattress knots increase initial biomechanical stability on a porcine cadaveric shoulder model. However, some clinical studies reporting structural defects on the medial of the footprint and on the musculotendinous junction in patients treated with double-row repair and TOE technique have led to doubts on the role of medial knots [25,26]. One of the most probable reasons of this insufficiency is the hypothesis of tendinous strangulation at the medial knot of the tendon–bone fixation. Tendon perforation, strong synthetic sutures and knots could disrupt tendinous microcirculation and lead to insufficiency with retears.

The results of the studies regarding the completely knotless TOE technique, which was developed to decrease medial insufficiency, are not clear either. In light of the studies demonstrating the importance of the medial row knots, it is probable that the knotless TOE has worse biomechanical properties compared to knotted medial rows [23,24]. However, Spang et al. have found the knotless TOE technique biomechanically similar to the conventional TOE on an ovine cadaveric study [27]. In a study by Burkhart et al. [28], which has been carried out on human cadaveric models, conventional TOE and completely knotless TOE techniques have demonstrated similar stability as well.

Although the improvement of biomechanical properties could assist in tendon–bone healing, current biomechanical studies fall short on replicating the rotator cuff repair model used in clinical practice and can only provide information about the initial biomechanics, being unable comment regarding the long-term stability of the repair. More importantly, in clinical setting, the torn tendon tissues generally demonstrate degeneration and fatty atrophy and probably have different characteristics compared to the cadaveric tendon tissue. Moreover, it is not known what the optimum pressure on the tendon–bone contact area is, which pressure stimulates healing and which pressure leads to tissue malnutrition and impairment of the healing process.

### Healing and retear

While the retear rates following arthroscopic repair can vary widely, rates approaching 94% have been reported in the literature for large and massive tears [1]. However, retears detected after surgery are generally asymptomatic and they are thought not to contribute significantly to the functional outcomes [29,30]. In contrast, some studies have reported findings which indicate that retears can impact functional healing [31,32]. In the literature, there are numerous studies investigating the structural integrity and retear rates of surgical techniques via methods such as magnetic resonance imaging (MRI), computerized tomography (CT) and ultrasonography (USG) during clinical follow-ups. Although there are studies concluding that double-row repair has no significant advantages over single-row repair in this aspect as well, the general consensus in the literature is that the biochemical superiorities of double-row repair demonstrated in experimental environments carry over to healing capacity and lead to lower retear rates.

The retrospective study of Sugaya et al. [6] is one of the longest follow-up studies comparing the retear rates of single and double-row repairs. The authors have detected retear rates of 56% in patients treated with single-row repairs and 27% in patients treated with double-row repairs after 3 years of follow-up and reported that this difference is statistically significant. In their large patient series, Mihata et al. [33] have reported a retear rate of 10.8% among their 65 single-row repair patients, 26.1% among their 23 double-row repair patients and 4.7% among their 107 TOE repair patients, at the end of 38.5 months. In the large and massive tear subgroup, the retear rate in the TOE repair group was 7.5%, while it was 62.5% in the single repair group and 41.7% in the double-row repair group. They have demonstrated that TOE repair has a significantly reduced rate of reruptures in large and massive tears. Charousset et al. [34] have investigated the retears of the patients using CT arthrography and demonstrated that anatomic healing was better with double-row repairs compared to single-row repairs. Tudisco et al. [35] have examined the radiological outcomes of single and double-row repairs in medium-sized rotator cuff tears by using MR arthrography and detected lower retear rates in double-row repairs. In their USG study, Gartsman et al. [36] have reviewed their patients who were treated by single-row repair or TOE repair due to the anteroposterior rotator cuff tears smaller than 25 mm. They have detected that among the patients treated by single-row repair, 30 patients out of 40 had their rotator cuff tears healed and among the patients treated by TOE repair, 40 patients out of 43 had their tears healed and concluded that TOE repair provides statistically significant superior healing. Pennington et al. [37] have evaluated their patients using MRI in their 132-patient series and found lower retear rate in patients treated by TOE technique compared to those treated by single-row repair. In a recent prospective comparative study by Hantes et al. [38], double-row constructs provided superior tendon healing compared to single row, in MRI assessments of medium to large rotator cuff tears at average 46 months follow-up.

As in the case of biomechanical studies, the literature is not fully in accordance regarding retear rates. In their randomized controlled study, Franceschi et al. [39] investigated the retear rates of large and massive rotator cuff tears by MRI at the end of 2 years of follow-up in their series of 60 patients and found no difference between the TOE technique and single-row repair. In conclusion, they claimed that double-row repair had no advantage in terms of creating an anatomical footprint over single-row repair. In a prospective randomized study, Ma et al. [40] evaluated their 53 patients treated with either single- or double-row rotator cuff repairs without considering the tear sizes after 2 years of follow-up and detected no significant difference in rotator cuff integrity. In the study in which Burks et al. [41] evaluated the results of medium-sized rotator cuff tears, they detected a retear rate of 10% in both groups at the end of 1 year after single- or double-row repairs. In another recent retrospective study, Kakoi et al. [42] couldn't detect any difference between the retear rates of single- and double-row constructs at average 16 months follow-up.

Some authors have tried to attain results with higher level of evidence about the effect of repair technique on retears by systematic reviews and meta-analyses. Duquin et al. [43], in their systematic review of 23 articles and 1252 patients, have detected a recurrence rate of 19% after single-row repairs and a rate of 7% after double-row repairs among tears smaller than 3 cm. Among tears larger than 3 cm, the rates of recurrence rose to 45% after single-row repairs and to 26% after double-row repairs. Saridakis and Jones [30] included six comparative studies with at least a level III of evidence in their systematic review and have come to a similar conclusion and claimed that double-row repairs provide better structural healing. Chen et al. [44], in their meta-analysis of levels I–III of evidence, have found that tendon healing is better in double-row repair compared to single-row repair, especially among tears larger than 3 cm. Millet et al. [45], in their meta-analysis where they included seven studies with only level I of evidence, have found higher retear rates among single-row repairs (25.9%) compared to double-row repairs (14.2%). Ying et al. [46] included seven randomized controlled studies and four prospective cohort studies in their meta-analysis and have detected that tendon healing was better and recurrence was lower after double-row repair among tears greater than 3 cm. Xu et al. [47], in their meta-analysis where they included nine studies, four of which being prospective randomized controlled studies, concluded that double-row repair had lower retear rates compared to single-row repair. Although DeHaan et al. [7] found a trend of double-row repairs to have lower rates of recurrence in their systematic analysis where they included seven studies with levels 1–II of evidence, this difference was not found to be statistically significant.

Although there are many comparative studies between single- and double-row repairs, the number of studies with high level of evidence comparing different double-row repair techniques is low. Hein et al. [48], in their systematic review of 32 studies, found lower retear rates in double-row and TOE techniques compared to single-row at the end of 1 year follow-up, among almost all tear sizes, but found no difference between double-row and TOE techniques in terms of retear rates. Kim et al. [49], in their prospective study, included 26 patients in each group for double-row repair and TOE repair and found no difference in terms of retear rates between the groups at the end of 2-year follow-up.

While better retear rates were obtained in comparison to single-row repair, both double-row and TOE repair techniques were also associated with high retear rates approaching 64%, especially among large and massive tears [6,50]. It is probable that the anchor overcrowding and the medial row knots could disrupt tendon vascularity and lead to an increased likelihood of a retear. Christoforetti et al. [8], in their clinical study comprising of 18 patients, investigated the effect of the TOE technique on tendon vascularity by using a doppler flowmetry probe and detected a 44.67% reduction in tendon blood flow after the repair is performed.

The evidences regarding the retear rates of the knotless TOE technique are few (Table 1). Although it has been reported that this technique could be prone to some complications such as suture slippage and loosening [51,52], some other studies found lower retear rates compared to the classical TOE technique [53–55]. Boyer et al. [53], in their series of 73 patients, evaluated the rotator cuff using MRI or CT arthrography. The authors found retear rates of 17.1% in knotless TOE group and 23.4% in conventional double-row repair group, but this difference was not found to be statistically significant. Rhee et al. [54] detected statistically lower rates of retear in the knotless TOE group (5.9%) compared to the knotted group (18.6%) in their series of 110 patients. The authors detected that 72.7% of the reruptures in the knotted group were located at the musculotendinous junction but detected no medial rotator cuff tears in the knotless group and proposed that knotless TOE repair could be superior in this regard as well. Millett et al. [55], in their up-to-date study where they evaluated 155 patients by MRI at the end of 2 years, found statistically lower rates of retear in the knotless TOE repair group compared to knotted TOE group.

**Table 1:** Summary of studies comparing two different double-row constructs.

	Study design	Repair type	*N*	Follow-up	Relevant findings
Spang et al. (2009) [27]	Cadaveric (ovine)	TOE vs. Knotless TOE	10 fresh frozen cadavers in each group	–	No significant difference between two constructs
Nassos et al. (2012) [22]	Cadaveric (human)	TOE vs. Knotless TOE	6 fresh frozen cadavers in each group	–	TOE repair technique best prevents leakage onto the rotator cuff footprint compared with knotless TOE repairs
Busfield et al. (2008) [23]	Cadaveric (human)	TOE vs. Knotless TOE	6 fresh frozen cadavers in each group	–	The addition of a knotless medial row compromises the construct leading to greater gapping and failure at lower loads
Burkhart et al. (2009) [28]	Cadaveric (human)	Double Row vs. Knotless TOE	7 fresh frozen cadavers in each group	–	Similar yield loads, ultimate loads, and cyclic displacements between two constructs
Hein et al. (2015) [48]	Systematic review	Double Row vs. TOE	32 studies; 1353 repairs	Minimum 1 year	No differences in retear rates were found
Kim et al. (2012) [49]	Retrospective comparative study	Double Row vs. TOE	26 patients in each group	Average 33 months (range, 10–54)	Comparable patient satisfaction, functional outcome, and rates of retear between two constructs
Rhee et al. (2012) [54]	Retrospective comparative study	TOE vs. Knotless TOE	59 patients in TOE, 51 patients in Knotless TOE group	Average 22 months (range, 12–34)	Similar clinical results between two constructs. However, the knotless group had a significantly lower retear rate compared with the conventional knot-tying group
Millett et al. (2017) [55]	Retrospective comparative study	TOE vs. Knotless TOE	155 shoulders in 151 patients	Average 2.9 years (range 2.0–5.4 years)	The repair technique did not affect the final functional outcomes, but patients with Knotless TOE were less likely to have a full-thickness rotator cuff retear
Boyer et al. (2015) [53]	Prospective comparative study	TOE vs. Knotless TOE	38 patients in TOE, 35 patients in Knotless TOE group	Average 29 months (range, 23–32)	Both bridging repair techniques achieved successful functional outcomes. In terms of structural outcome, the knotless TOE construct showed a lower but not significant retear rate

### Clinical outcomes

Although double-row repair techniques yield superior results compared to single-row techniques in regard to biomechanical properties and retear rates in conducted experiments and clinical studies, there are question marks regarding the contribution of these advantages to functional outcomes. Many prospective clinical trials have reported that single- and double-row repairs show no differences in terms of functional outcomes [39,41,56–58]. However, especially for large and massive tears, the number of studies reporting better functional outcomes with double-row repairs cannot be ignored.

Park et al. [59], in their series of 78 patients, found that double-row repair yielded better functional outcomes (Constant and ASES) compared to single-row repair in large and massive tears (>3 cm) at the end of 2 years of follow-up, and that the functional outcomes were similar in small and medium-sized tears. Denard et al. [60], in their retrospective study, detected good or perfect functional outcomes (UCLA) in 90% of double-row repairs and 70.9% of single-row repairs in massive rotator cuff tears at the end of an average 99 months follow-up and proposed that double-row repair should be preferred when there's adequate tendon mobility. In 3–5 cm sized tears, Carbonel et al. [61] found the functional scores (UCLA and ASES) and abduction-external rotation forces of double-row repair to be significantly better than those of single-row repair at the end of 2 years follow-up. Additionally, range of motion and internal–external rotation forces were found to be better in double-row repair among 1–3 cm sized tears as well, but no significant difference was detected in regard to functional scores.

Although meta-analyses generally report better outcomes with double-row repairs compared to single-row repairs in relation to retear rates, functional outcomes were generally found similar. Chen et al. [44], in their meta-analysis, detected no difference in terms of clinical outcomes between single-row and double-row repairs, even though they found higher retear rates with single-row repair. Millett et al. [45] found no significant difference between the two techniques in terms of functional outcomes (ASES, UCLA and Constant) even though they demonstrated higher rerupture rates among single-row repairs compared to double-row in their meta-analysis. Similarly, DeHaan et al. [7] found similar functional outcomes and complication rates between the two techniques. Sheibani-Rad et al. [62], in their meta-analysis which included five studies with level I of evidence, found no functional difference between single- and double-row repairs. On the other hand, Xu et al. [47] detected a higher ASES functional score and better internal rotation in double-row repairs compared to single-row repairs. However, they detected no difference in terms of constant and UCLA scores, forward flexion, external rotation or muscle strength. In their prospectively designed study, Hantes et al. [38] couldn't detect any functional difference between single and double-row repairs, despite the superior tendon healing in double-row group. Jeong et al. [63] also reported similar functional outcomes between single-row repair and TOE repair in a large retrospective cohort study including 415 patients.

Although many studies reported perfect functional outcomes in knotted and knotless TOE techniques [53,64,65], the number of studies in which they are compared with conventional double-row repairs is negligibly low (Table 1). Kim et al. [49], in their study with level II of evidence, detected no difference in terms of rertear rates and functional outcomes between double-row repair and the TOE technique at the end of 2 years of follow-up among tears sized 1–4 cm. Boyer et al. [53], in their series of 73 patients, detected no functional differences between knotted and knotless TOE techniques. Millett et al. [55] obtained similar functional outcomes with knotted and knotless TOE techniques at the end of 2 years of follow-up in their series of 155 patients. However, there is currently not enough clinical data to determine the functional advantages of various double-row technique modifications over each other.

## Discussion

The principle aspects that can affect functional outcomes in the treatment of rotator cuff tears are the surgical technique, patient selection, rehabilitation protocol and the microenvironment of healing. Among these, the one that draws the most attention and therefore has the greatest presence in the literature is the surgical technique. By modifying various parameters such as the number of suture anchors, locations and suture configurations, clinical outcomes are sought to be improved. Upon a general glance at the literature, the biomechanical superiority of double-row repairs has been proven. Among these properties are increased repair strength, reduced gap formation and a wider footprint contact. The TOE technique also appears to be biomechanically promising. It is thought that it can positively impact healing, especially through preventing synovial fluid leakage into the healing zone and increasing tendon–bone contact pressure. However, there is as of yet not enough evidence on whether an increased pressure at the tendon–footprint interface can assist in healing. As likely as an increased contact pressure is to stimulate healing, it is also probable that it can disrupt the biological environment of the tendon and reduce tendon bone healing.

In regard to retear rates, both double-row and TOE techniques have yielded better outcomes compared to single-row repair. Although there exist conflicting results regarding clinical outcomes, there are many studies indicating that double-row repair techniques could be more advantageous especially for tears larger than 3 cm. Most of the studies and meta-analyses that failed to detect a functional difference between double-row repairs and single-row repairs have evaluated tear sizes all together. However, as Lorbach et al. [66] have proposed, the size of the tear is an independent factor in relation to functional outcomes and evaluating all tear sizes together while comparing the techniques would make it more difficult to detect differences in clinical outcomes.

Although there are many studies that report no meaningful correlation between retear rates and functional outcomes [67,68], Gazielly et al. [69], Sugaya et al. [6] and Huijsmans et al. [50] have detected meaningful correlations between postoperative repair integrity and functional outcomes. Mihata et al. [33] have also detected that the functional outcomes of patients who had developed retears was worse in their large patient series. Asymptomatic tears in the mid-term follow ups could lead to larger and symptomatic tears that require revision surgery later on. It is probable that a better structural integrity could yield better functional outcomes in the long term. Mall et al. [70], in their studies where they evaluated 195 asymptomatic rotator cuff tear patients, detected that 23% of the patients eventually became symptomatic within two years after being included in the study. Yamaguchi et al. [71] followed up on 45 asymptomatic rotator cuff tear patients and reported that 51% of the patients became symptomatic within 2.8 years of being included in the study. For this reason, longer follow-up durations could facilitate the investigation of the fates of retears that are more often encountered in patients treated with single-row repairs.

Although single-row repair is regarded as adequate for small-medium sized tears, double-row and TOE repair techniques could be superior for providing structural integrity in large and massive tears. However, studies in the literature that fail to find a meaningful functional difference between transosseous, single-row, double-row and TOE techniques cause conflict, and the cost-effectiveness of double-row repairs should be questioned due to the increased number of anchors and the prolonged intraoperative time. It is important to optimize the costs without jeopardizing the treatments of the patients. Especially the interventions that are thought to yield comparable functional outcomes have to be compared in regard to cost-effectiveness. In general, although double-row repairs are more costly procedures, the functional outcomes, repetitive interventions and diagnostic tests must also be put into consideration in order to determine cost-effectiveness. In this regard, the number of studies investigating cost-effectiveness in the treatment of rotator cuff tears is negligibly low. Bisson et al. [72] analyzed the costs of single- and double-row repairs. They suggested that double-row repair might decrease the costs by decreasing the revision rates with better healing capacity but couldn't come up with a scientific evidence. Genuario et al. [73], in their study where they compared single-row and double-row repairs in terms of cost, determined that double-row repairs are not as cost-effective as single-row repairs. Huang et al. [74], in their study based on the Canadian health care system, found the cost-effectiveness of double-row repairs to be better than single-row repairs when they included additional costs such as revision operations and additional imaging tests. In a recent study by Chalmers et al. [75], the authors reported higher costs with single-row repairs compared to double-row, but the single-row repairs in this study were made by triple-loaded anchors which require more time for suture passage and knot tying than double-row repair, explaining the increased cost. Therefore, they concluded that these findings cannot be generalizable to other surgeons with different indications for different repair patterns. There is need for more studies in the setting of different health care systems for this matter.

## Conclusions

Techniques regarding rotator cuff repairs are advancing constantly. In conclusion, in light of current literature, we believe that it is possible to obtain effective treatment for rotator cuff tears smaller than 1 cm using single-row repair. For tears between 1–3 cm, there is no clear consensus in current literature and in our opinion, any of the single-row, double-row, knotted TOE or knotless TOE techniques can be chosen depending on the preference of the surgeon and the expectation of the patient. For tears larger than 3 cm, we believe that the TOE technique will yield better outcomes both functionally and in terms of repair integrity. Although identification of the tear pattern is one of the most important guiding steps in choosing a repair technique, the surgeon must not forget to evaluate each patient separately, and when a decision regarding surgical technique is to be made, factors such as age, activity level and functional expectation must be considered. Future studies with high levels of evidence could guide us better.

## Conflict of interest

The authors declare that they have no conflict of interest in relation to this article.
